# Ultrasound Appearance of Intravascular Uterine Smooth Muscle Tumor of Uncertain Malignant Potential (STUMP): A Case Report

**DOI:** 10.3390/diagnostics14131438

**Published:** 2024-07-06

**Authors:** Nina Montik, Camilla Grelloni, Alessandra Filosa, Gaia Goteri, Jacopo Di Giuseppe, Leonardo Natalini, Andrea Ciavattini

**Affiliations:** 1 Woman’s Health Sciences Department, Gynecologic Section, Polytechnic University of Marche, 60123 Ancona, Italy; nina.montik@ospedaliriuniti.marche.it (N.M.); c.grelloni@pm.univpm.it (C.G.); jacopo.digiuseppe@ospedaliriuniti.marche.it (J.D.G.); l.natalini@pm.univpm.it (L.N.); 2 Anatomic Pathology, Department of Biomedical Sciences and Public Health, Polytechnic University of Marche, 60126 Ancona, Italy; alessandra.filosa@ospedaliriuniti.marche.it (A.F.); g.goteri@staff.univpm.it (G.G.)

**Keywords:** uterine smooth muscle tumor of uncertain malignant potential, STUMP, intravascular leiomyomatosis, IVL, parauterine mass, pelvic ultrasound

## Abstract

A 43-year-old patient with a history of uterine fibromatosis was referred to our hospital for menometrorrhagia and pelvic pain. At the pelvic ultrasound, a highly-vascularized myometrial lesion in volumetric increase was described. An elongated, solid, hypoechoic, painless, and highly vascularized left parauterine mass was identified. On histological examination, a uterine smooth muscle tumor of uncertain malignant potential (STUMP) with intravascular invasion of the left uterine vein was diagnosed. The adnexa and peritoneum were free of disease. On a retrospective evaluation of the ultrasound images, we noticed that the intravascular lesion showed sonographic features comparable to the original mass. Moreover, the Color Doppler (CD) analysis revealed an interrupted blood flow within the left uterine vein. In this case, the ultrasound proved to be an accurate diagnostic tool. When inhomogeneous uterine masses are suspected, and a parauterine/paraadnexal mass surrounded by irregular vessels are identified, the sonographer should take into account a risk of intravascular invasion. The patency of uterine and ovarian vessels should be accurately evaluated, to guide a tailored patient surgical approach.

## 1. Introduction

The World Health Organization (WHO) defines a STUMP as a smooth muscle tumor which, based on criteria of necrosis, atypia, and mitosis, cannot be unequivocally diagnosed as a leiomyoma (LM) or leiomyosarcoma (LMS) [1]. The preoperative diagnosis of a smooth muscle tumor of uncertain malignant potential (STUMP) is challenging as it has no specific radiological features [2].

Intravenous leiomyomatosis (IVL) is a rare benign smooth muscle tumor with malignant potential that arises from myometrial masses and can extend through the uterine or ovarian veins. 

The STUMP shows two peaks of incidence in the fertile and postmenopausal period, with an average age of 41–48 years, as our patient [1,3]. The symptoms are similar for LM and LMS [3]. The STUMP presents a low malignant potential, but the risk of recurrence varies from 0 to 36% [3]. The uterus is the most frequent site of relapse (60%), while in the rest of cases it occurs elsewhere, in the lungs or abdomen. The recurrence can rarely present as a malignant transformation into LMS or liposarcomas, worsening the prognosis [2]. Recent data showed that the most influential factor on the risk of STUMP recurrence was unprotected morcellation, either during demolition surgery or a myomectomy [2]. 

Therefore, an accurate diagnostic evaluation of uterine masses and adequate surgical programming are mandatory to avoid surgical procedures that may worsen the prognosis and, in the case of IVL, make it possible to decide whether to remove the lesions.

This case report presents a unique case of intrasurgical, sonographic, and histological images of the intravenous extension of a STUMP.

## 2. Case Presentation

**Figure 1:**
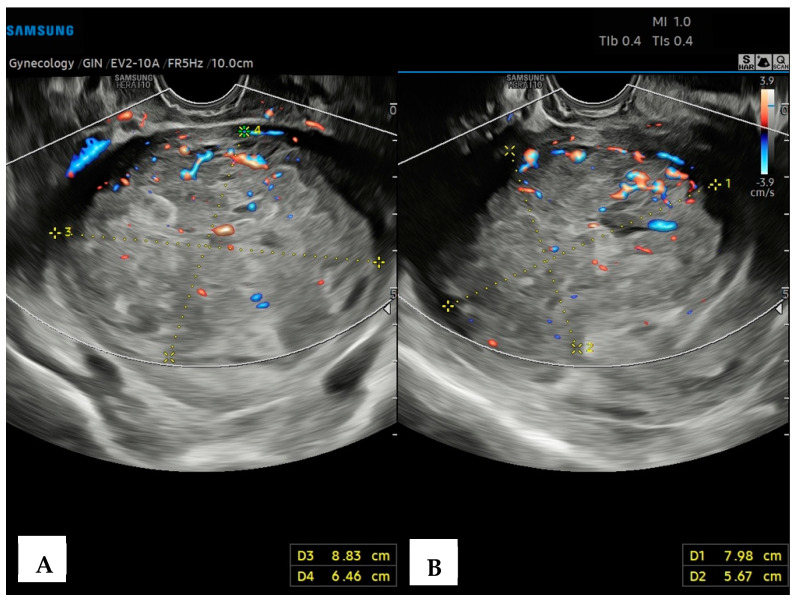
Transvaginal ultrasound with Color Doppler analysis. (**A**) Longitudinal plane; (**B**) axial plane showing a hypoechoic, heterogeneous uterine mass, without calcifications or acoustic shadows, measuring (L × H × W) 8.8 × 7.9 × 6.4 cm. The Color Doppler analysis reveals an abundant perilesional and intralesional vascularization.

A 43-year-old healthy woman was referred to our Hospital in September 2022, following menometrorrhagia, pelvic pain and a recent diagnosis of uterine fibromatosis. Informed written consent for the publication of data was obtained. An expert transvaginal ultrasound with the Samsung HeraI10 was performed, revealing an enlarged, globose-shaped uterus, with a transmural (FIGO 3-5) uterine mass of the posterior body–fundus wall, which was hypoechoic, non-homogeneous, and without calcifications, cystic areas, or acoustic shadows, measuring 8.8 × 7.9 × 6.4 cm, and highly vascularized (Color Score 3-4, CS3-4), at the Color Doppler (CD) analysis (Figure 1). 

**Figure 2:**
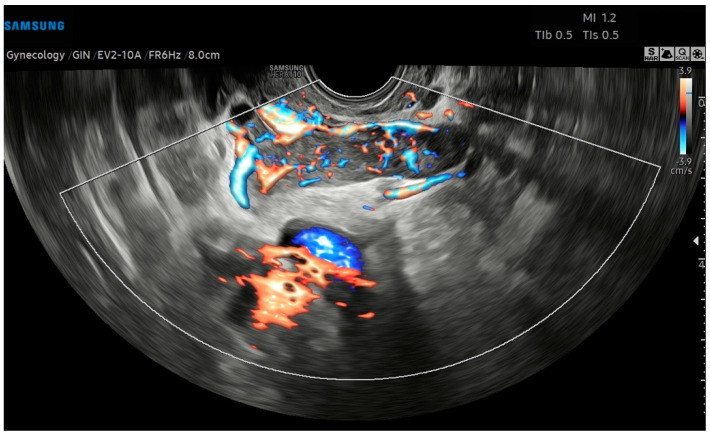
Transvaginal ultrasound with Color Doppler analysis; the longitudinal plane shows a solid elongated, hypoechoic, left parauterine mass. The Color Doppler analysis emphasizes the intense vascularization of the lesion.

The preoperatory hypothesis was an atypical myoma or adenomyoma, in volumetric growth >50% compared to the previous control 3 months earlier. The adnexa, pouch of Douglas, pelvic peritoneum, and the pelvic tract of ureters were regular. On the left parauterine side, an elongated solid hypoechoic formation, with defined contours, abundant intralesional vascularization on the CD image (CS4), and extension toward the ovarian fossa, measuring 6.7 × 1.6 × 2.5 cm, was described (Figure 2). Pelvic Magnetic Resonance Imaging (RMI) confirmed the atypical myometrial lesion, without parauterine nor adnexal anomalies. Serum tumor markers (LDH, CEA, CA125, CA19.9, He4) were normal.

**Figure 3:**
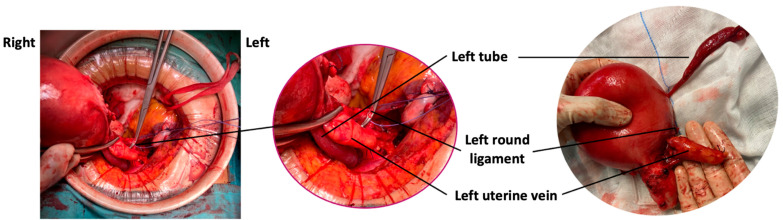
The intrasurgical observation reveals a normal left tube and an enlarged left uterine vein, filled with friable whitish material, dissected at its entrance into the ipsilateral internal iliac vein.

Due to the atypical appearance of the uterine mass, a laparotomy with a total hysterectomy and bilateral salpingectomy was performed. During surgery, the left uterine vein presented with an abnormal volume and consistency, corresponding to the parauterine mass previously identified from the ultrasound (Figure 3). The entire vessel was removed by sectioning it at its origin from the internal iliac vein prior to the hysterectomy. 

The frozen section pathology of the parauterine mass was performed during surgery. The anatomopathologist reported the presence of a leiomyoma with growth within the vein.

**Figure 4:**
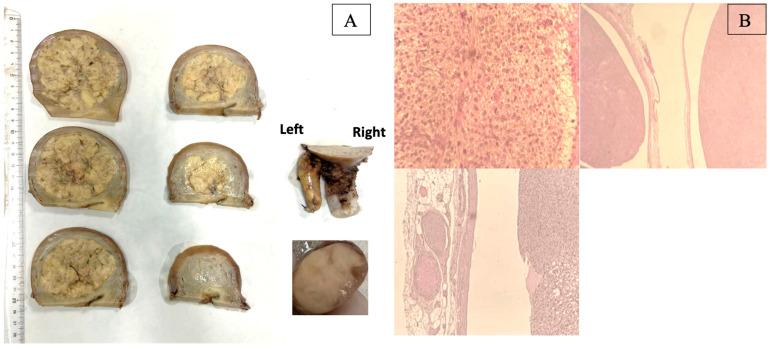
Gross appearance of the tumor after fixation in formalin (**A**): enlarged uterus due to the presence of a yellowish nodule measuring 8.0 × 7.0 × 7.0 cm, within which a vascular structure is visible containing the same yellowish fasciculated proliferation. Another elongated yellowish intravascular nodule measuring 5.0 × 2.0 × 1.0 cm emerges laterally from the cervix. Microscopic examination (**B**): myometrial mesenchymal proliferation composed of monomorphic spindle cells, with noninfiltrative growth margins. No evidence of cytological atypia and necrosis. Mitosis 8–10/high power field. The cells that constitute the lesion show signs of intravascular venous invasion.

The definitive histological examination of the uterine mass showed a smooth muscle cell tumor of uncertain malignant potential, with intravascular extension into the left uterine vein (Figure 4).

The retrospective analysis of the preoperatory ultrasound images revealed that the parauterine lesion presented sonographic features similar to the uterine tumor. Video recordings showed a completely interrupted blood flow on the CD image in the left uterine vein. At the time of preoperative examinations, the patient did not have any clinical or ultrasound evidence of pelvic congestion nor significant right parauterine vein dilatation. After surgery, the extrapelvic dissemination of the disease was excluded by a chest–abdominal Computerized Tomography (CT) scan with contrast, CT angiography of the thoraco-abdominal aorta, and echocardiography. The patient underwent therapy with Leuprolide acetate at a dose of 11.25 mg intramuscularly for 6 months; she is under follow-up with TV-US and chest–abdomen CT scan every 6 months, and she has always tested negative for the spread/recurrence of disease.

## 3. Discussion

Several retrospective studies made efforts to describe specific and reproducible STUMP sonographic features, with conflicting results. One study group described mixed echogenicity, regular borders, and microcystic internal areas, while calcifications and acoustic shadows were rare findings [4]; others described multiple, hypoechoic, non-cystic uterine tumors [5]. The CD study usually reveals a highly vascularized lesion, with a circumferential or intralesional blood flow pattern [4,6]. This makes the STUMP sonographically indistinguishable, with the risk of equivocal diagnosis with LM, LMS, atypical leiomyomas, and adenomyomas. The presence of central coagulative necrosis on the contrast-enhanced MRI (CE-MRI) as well as the “hollow ball sign” on the Positron Emission Tomography [PET] scan showed good specificity for the differential diagnosis between STUMP/LMS [7,8]. The confusing clinical aspects and the poor reliability of preoperative imaging make the STUMP a lesion mainly diagnosed through histological analyses of specimens.

Intravenous leiomyomatosis (IVL) is a rare smooth muscle benign tumor with malignant behavior, occurring only in women. It generates from myometrial masses and can extend through the uterine or ovarian veins, to the renal vein and inferior vena cava. In 10% of cases, IVL invades the right side of the heart and receives the definition of intracardiac leiomyomatosis (ICL) [9]. In the early stages, IVL is confined to the pelvis and the clinical presentation is comparable to uterine fibroids, while in the later stages, it involves renal veins or the inferior cava, and can cause the swelling of lower extremities. In advanced stages, ICL reaches the right atrium, right ventricle, and pulmonary arteries, causing the congestion of right cardiac sections (dyspnea, syncope, heart failure, ascites, hepatosplenomegaly), pulmonary embolism, and, rarely, sudden cardiac death [10]. The MUSA consensus stated that IVL has no precise sonographic characteristics, resembling a uterine fibroid [11]. The authors of [12] described IVL as a hypoechoic tubular mass located in the pelvic or uterine veins, and which therefore can be confused with a myoma of the broad ligament, an adnexal neoformation, or venous thrombosis. The CD image usually shows an abundant blood flow [13]. CT and MRI scans have uncertain value, but they can differentiate between venous and pelvic masses. CT angiography can be used to excellently study the extension of IVL, especially in advanced stages [13].

As far as we know, this is the first article reporting intrasurgical, sonographic, and histological images of the intravenous extension of a STUMP. In our case, the left uterine vein appeared totally obliterated by the presence of the intravascular mass through the CD analysis. This lesion showed an echogenicity pattern and rich vascularization that is comparable to a uterine mass, while its shape was delimited by vessel walls. It is probable that intravascular extension is better detectable within venous vessels than arteries, due to the characteristics of the vascular walls and blood flow. The MRI scan did not identify anomalies of the uterine vascular plexus and there was no indication to perform CT angiography before surgery. The ultrasound technique with the CD analysis might have a good diagnostic performance. 

## 4. Conclusions

In summary, this case report of an intravenous extension of a STUMP has highlighted the diagnostic approach taken for this rare condition, emphasizing the need for an expert sonographer’s preoperative evaluation to ensure optimal patient management.

In patients with suspected uterine mesenchymal tumors, the sonographic detection of elongated solid parauterine/paraadnexal masses surrounded by irregular vessels and resembling original uterine lesions, should be accurately evaluated with Color and Power Doppler images, to exclude vessels lumen involvement. The preoperative evaluation of the severity of myomatous intravascular extension is essential for correct surgical planning and the adequate modulation of the radicality.

## Data Availability

The data presented in this study are available on request from the corresponding author due to patient privacy.
